# Supplementary material for: Critical transitions in the Amazon forest system
Source: Nature. 2024 Feb 14;626(7999):555–64. doi: 10.1038/s41586-023-06970-0 (PMC10866695; doi:10.1038/s41586-023-06970-0)
Supplement: Supplementary file 1 — Supplementary Information [file 41586_2023_6970_MOESM1_ESM.docx]

**Supplementary Information for**

**[Critical transitions in the Amazon forest system]**

Bernardo M. Flores^1^*, Encarni Montoya^2^, Boris Sakschewski^3^, Nathália Nascimento^4^, Arie Staal^5^, Richard A. Betts^6,7^, Carolina Levis^1^, David M. Lapola^8^, Adriane Esquível-Muelbert^9^, Catarina Jakovac^10^, Carlos A. Nobre^4^, Rafael S. Oliveira^11^, Laura S. Borma^12^, Da Nian^3^, Niklas Boers^3,13^, Susanna B. Hecht^14^, Hans ter Steege^15,16^, Julia Arieira^17^, Isabella L. Lucas^18^, Erika Berenguer^19^, José A. Marengo^20^, Luciana V. Gatti^12^, Caio R. C. Mattos^21^ & Marina Hirota^1,11,22^*

**Affiliations**

1. Graduate Program in Ecology, Federal University of Santa Catarina, Florianopolis, Brazil.
2. Geosciences Barcelona, Spanish National Research Council, Barcelona, Spain.
3. Potsdam Institute for Climate Impact Research, Member of the Leibniz Association, Potsdam, Germany.
4. Institute of Advanced Studies, University of São Paulo, São Paulo, Brazil.
5. Copernicus Institute of Sustainable Development, Utrecht University, Utrecht, The Netherlands.
6. Met Office Hadley Centre, Exeter, UK
7. Global Systems Institute, University of Exeter, UK
8. Center for Meteorological and Climatic Research Applied to Agriculture, University of Campinas, Campinas, Brazil.
9. School of Geography, Earth and Environmental Sciences, University of Birmingham, Birmingham, UK. Birmingham Institute of Forest Research, University of Birmingham, UK.
10. Department of Plant Sciences, Federal University of Santa Catarina, Florianopolis, Brazil.
11. Department of Plant Biology, University of Campinas, Campinas, Brazil.
12. Division of Impacts, Adaptation and Vulnerabilities (DIIAV), National Institute for Space Research, São José dos Campos, Brazil.
13. Earth System Modelling, School of Engineering and Design, Technical University of Munich, Munich, Germany.
14. Luskin School for Public Affairs and Institute of the Environment, University of California, Los Angeles, USA.
15. Naturalis Biodiversity Center, Leiden, The Netherlands.
16. Quantitative Biodiversity Dynamics, Utrecht University, Utrecht, The Netherlands.
17. Science Panel for the Amazon- SPA, São José dos Campos, SP, Brazil.
18. Sustainable Development Solutions Network, New York, USA.
19. Environmental Change Institute, University of Oxford, Oxford, UK.
20. Centro Nacional de Monitoramento e Alerta de Desastres Naturais, São José dos Campos, Brazil.
21. Program in Atmospheric and Oceanic Sciences, Princeton University, Princeton, NJ, USA
22. Department of Physics, Federal University of Santa Catarina, Florianopolis, Brazil.

* Corresponding authors. Email: mflores.bernardo@gmail.com, marinahirota@gmail.com

**Table 1**. Models from the 6^th^ Phase of the Coupled Model Intercomparison Project (CMIP6) used in the study.

| **Model** | **Dynamic vegetation** |
| --- | --- |
| ACCESS-CM2 | No |
| ACCESS-ESM1-5 | No |
| AWI-CM-1-1-MR | No |
| BCC-CSM2-MR | No |
| CAMS-CSM1-0 | No |
| CAS-ESM2-0 | No |
| CESM2-WACCM | No |
| CMCC-CM2-SR5 | No |
| CMCC-ESM2 | No |
| CNRM-CM6-1 | No |
| CNRM-CM6-1-HR | No |
| CNRM-ESM2-1 | No |
| EC-Earth3 | No |
| EC-Earth3-CC | No |
| EC-Earth3-Veg | Yes |
| EC-Earth3-Veg-LR | No |
| FGOALS-f3-L | No |
| FIO-ESM-2-0 | No |
| GFDL-CM4 | No |
| GFDL-ESM4 | Yes |
| HadGEM3-GC31-LL | No |
| IITM-ESM | No |
| KACE-1-0-G | No |
| KIOST-ESM | No |
| MIROC6 | No |
| MPI-ESM1-2-HR | No |
| MPI-ESM1-2-LR | Yes |
| MRI-ESM2-0 | No |
| NESM3 | No |
| NorESM2-MM | No |
| TaiESM1 | Yes |
| UKESM1-0-LL | Yes |
| CESM2 | No |
