# Supplementary material for: Critical transitions in the Amazon forest system
Source: Nature. 2024 Feb 14;626(7999):555–64. doi: 10.1038/s41586-023-06970-0 (PMC10866695; doi:10.1038/s41586-023-06970-0)
Supplement: Supplementary file 2 — Reporting Summary [file 41586_2023_6970_MOESM2_ESM.pdf]

nature portfolio

Corresponding author(s): Bernardo M. Flores and Marina Hirota

Last updated by author(s): Nov 5, 2023

Reporting Summary

Nature Portfolio wishes to improve the reproducibility of the work that we publish. This form provides structure for consistency and transparency in reporting. For further information on Nature Portfolio policies, see our Editorial Policies and the Editorial Policy Checklist.

Statistics

For all statistical analyses, confirm that the following items are present in the figure legend, table legend, main text, or Methods section.

n/a	Confirmed	
☒	☐	The exact sample size (*n*) for each experimental group/condition, given as a discrete number and unit of measurement
☒	☐	A statement on whether measurements were taken from distinct samples or whether the same sample was measured repeatedly
☒	☐	The statistical test(s) used AND whether they are one- or two-sided<br>*Only common tests should be described solely by name; describe more complex techniques in the Methods section.*
☒	☐	A description of all covariates tested
☒	☐	A description of any assumptions or corrections, such as tests of normality and adjustment for multiple comparisons
☒	☐	A full description of the statistical parameters including central tendency (e.g. means) or other basic estimates (e.g. regression coefficient) AND variation (e.g. standard deviation) or associated estimates of uncertainty (e.g. confidence intervals)
☒	☐	For null hypothesis testing, the test statistic (e.g. *F*, *t*, *r*) with confidence intervals, effect sizes, degrees of freedom and *P* value noted<br>*Give P values as exact values whenever suitable.*
☒	☐	For Bayesian analysis, information on the choice of priors and Markov chain Monte Carlo settings
☒	☐	For hierarchical and complex designs, identification of the appropriate level for tests and full reporting of outcomes
☒	☐	Estimates of effect sizes (e.g. Cohen's *d*, Pearson's *r*), indicating how they were calculated

Our web collection on statistics for biologists contains articles on many of the points above.

Software and code

Policy information about availability of computer code

Data collection	No software was used.
Data analysis	Potential analysis - Using potential analysis (Livina et al. 2010), an empirical stability landscape was constructed based on spatial distributions of tree cover against mean annual precipitation (MAP), maximum cumulative water deficit (MCWD) and dry season length (DSL). Here we followed the methodology of Hirota et al. (2011). For bins of each of the variables, the probability density of tree cover was determined using the MATLAB function ksdensity with a bandwidth of 5%. We applied Gaussian weights to the variable with a standard deviation of 0.05 times the range of the variable: 0-3500 mm/yr for MAP, 0-12 months for DSL and -800-0 mm for MCWD. Local maxima of the resulting probability density function are considered to be stable states, in which local maxima below a threshold value of 0.003 were ignored.<br><br>Atmospheric moisture tracking - To determine the atmospheric moisture flows between the Amazonian countries, we use the Lagrangian atmospheric moisture tracking model UTrack (Tuinenburg & Staal 2020). The model tracks the atmospheric trajectories of parcels of moisture, updates their coordinates at each time step of 0.1 h and allocates moisture to a target location in case of precipitation. For each mm of evapotranspiration, 100 parcels are released into the atmosphere. Their trajectories are forced with evaporation, precipitation, and wind speed estimates from the ERA5 reanalysis product at 0.25º horizontal resolution for 25 atmospheric layers (Hersbach et al. 2020). Here we use the runs from Tuinenburg et al. (2020), who published monthly climatological mean (2008–2017) moisture flows between each pair of 0.5º grid cells on Earth. Here we aggregated these monthly flows, resulting in mean annual moisture flows between all Amazonian countries during 2008–2017. For more details of the model runs, we refer to Tuinenburg & Staal (2020) and Tuinenburg et al. (2020).

For manuscripts utilizing custom algorithms or software that are central to the research but not yet described in published literature, software must be made available to editors and reviewers. We strongly encourage code deposition in a community repository (e.g. GitHub). See the Nature Portfolio guidelines for submitting code & software for further information.

Data

Policy information about availability of data

All manuscripts must include a data availability statement. This statement should provide the following information, where applicable:

- Accession codes, unique identifiers, or web links for publicly available datasets
- A description of any restrictions on data availability
- For clinical datasets or third party data, please ensure that the statement adheres to our policy

All datasets used are publicly available, including the land-cover changes and Amazonian contour from MapBiomas Amazonian Project (2022, https://amazonia.mapbiomas.org), tree cover data from the Moderate Resolution Imaging Spectroradiometer (MODIS, https://modis.gsfc.nasa.gov), rainfall data from Climate Hazards Group InfraRed Precipitation with Station data (CHIRPS, https://www.chc.ucsb.edu/data/chirps), temperature data from Climatic Research Unit (CRU, https://www.uea.ac.uk/groups-and-centres/climatic-research-unit), and burnt area also from MODIS (MCD14ML, Collection 6).

Human research participants

Policy information about studies involving human research participants and Sex and Gender in Research.

Reporting on sex and gender	This type of information is not part of our study and therefore has not been collected.
Population characteristics	See above.
Recruitment	See above.
Ethics oversight	See above.

Note that full information on the approval of the study protocol must also be provided in the manuscript.

Field-specific reporting

Please select the one below that is the best fit for your research. If you are not sure, read the appropriate sections before making your selection.

☐ Life sciences ☐ Behavioural & social sciences ☒ Ecological, evolutionary & environmental sciences

For a reference copy of the document with all sections, see nature.com/documents/nr-reporting-summary-flat.pdf

Ecological, evolutionary & environmental sciences study design

All studies must disclose on these points even when the disclosure is negative.

Study description

Our study is a review of the existing evidence in the literature about the mechanisms that could cause a tipping point in the Amazon forest system, including evidence from modelling studies, paleorecords and observational studies on ecology and climatology. To highlight the most relevant findings, we re-analysed some of the data, focusing on the Amazonian region, using datasets that are publicly available.

Research sample

Our study site was the area of the Amazon basin, considering parts of the Brazilian Cerrado biome to the south, the Orinoco basin to the north, and eastern parts of the Andes to the west. We chose this contour to allow better communication with the MapBiomas Amazonian Project (2022, https://amazonia.mapbiomas.org). For specific interpretation of our results, we considered the contour of the current extension of the Amazon forest biome, which excludes surrounding tropical savanna biomes.

We used the Vegetation Continuous Fields (VCF) data from the Moderate Resolution Imaging Spectroradiometer (MODIS, https://modis.gsfc.nasa.gov/) for the year 2001 at 250-m resolution (DiMiceli et al. 2011) to analyze tree cover distributions in the Amazon basin.

We used the Climate Hazards Group InfraRed Precipitation with Station data (CHIRPS, https://www.chc.ucsb.edu/data/chirps) (Mitchell and Jones, 2005) to estimate mean annual rainfall and rainfall seasonality for the present across the Amazon basin, based on monthly means from 1981 through 2020, at a 0.05o spatial resolution.

We used the Climatic Research Unit (CRU, https://www.uea.ac.uk/groups-and-centres/climatic-research-unit) (Funk et al. 2015) to estimate mean annual temperature for the present across the Amazon basin, based on monthly means from 1981 through 2020, at a 0.5o spatial resolution.

To mask deforested areas until 2012, we used information from the MapBiomas Amazonia Project (2022), Collection 3, of Amazonian Annual Land Cover & Land Use Map Series, with the link: https://amazonia.mapbiomas.org.

To assess forest fires across the Amazon forest biome, we used burnt area fire data obtained from the AQUA sensor onboard the MODIS satellite. Only active fires with a confidence level of 80% or higher were selected. The data are derived from MODIS

	MCD14ML (Collection 6), available in FIRMS – Fire Information for Resource Management System. The data were adjusted to a spatial resolution of 1km, according to the methodology of Silva Junior et al., (2019).
Sampling strategy	Our sample sizes are limited by the spatial resolution of the datasets described above and by the area of the Amazon basin.
Data collection	Please, see response to "Research sample".
Timing and spatial scale	Our temporal and spatial scales are limited by the resolutions of the remote sensing datasets described above.
Data exclusions	No data were excluded by our analyses.
Reproducibility	We re-analyse previously published and openly available datasets, with focus on the Amazon forest.
Randomization	This is not relevant to our study.
Blinding	Blinding was not relevant to our study. We analysed all information within our study area.

Did the study involve field work? ☐ Yes ☒ No

Reporting for specific materials, systems and methods

We require information from authors about some types of materials, experimental systems and methods used in many studies. Here, indicate whether each material, system or method listed is relevant to your study. If you are not sure if a list item applies to your research, read the appropriate section before selecting a response.

Materials & experimental systems

n/a	Involved in the study
☒	☐ Antibodies
☒	☐ Eukaryotic cell lines
☒	☐ Palaeontology and archaeology
☒	☐ Animals and other organisms
☒	☐ Clinical data
☒	☐ Dual use research of concern

Methods

n/a	Involved in the study
☒	☐ ChIP-seq
☒	☐ Flow cytometry
☒	☐ MRI-based neuroimaging
